# Supplementary material for: Effect of Ginseng Sapogenin Protopanaxadiol-Enriched Rice (DJ-PPD) on Immunomodulation
Source: Plants (Basel). 2023 Feb 8;12(4):767. doi: 10.3390/plants12040767 (PMC9959806; doi:10.3390/plants12040767)
Supplement: Supplementary file 1 [file plants-12-00767-s001.zip › plants-2200471-supplementary.pdf]

Supplementary Materials

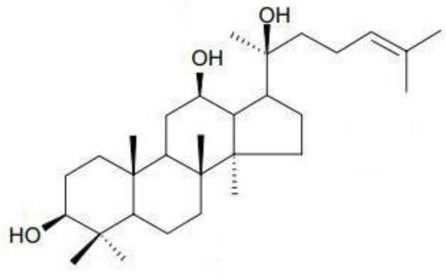

Figure S1. Protopanaxadiol chemical structure.

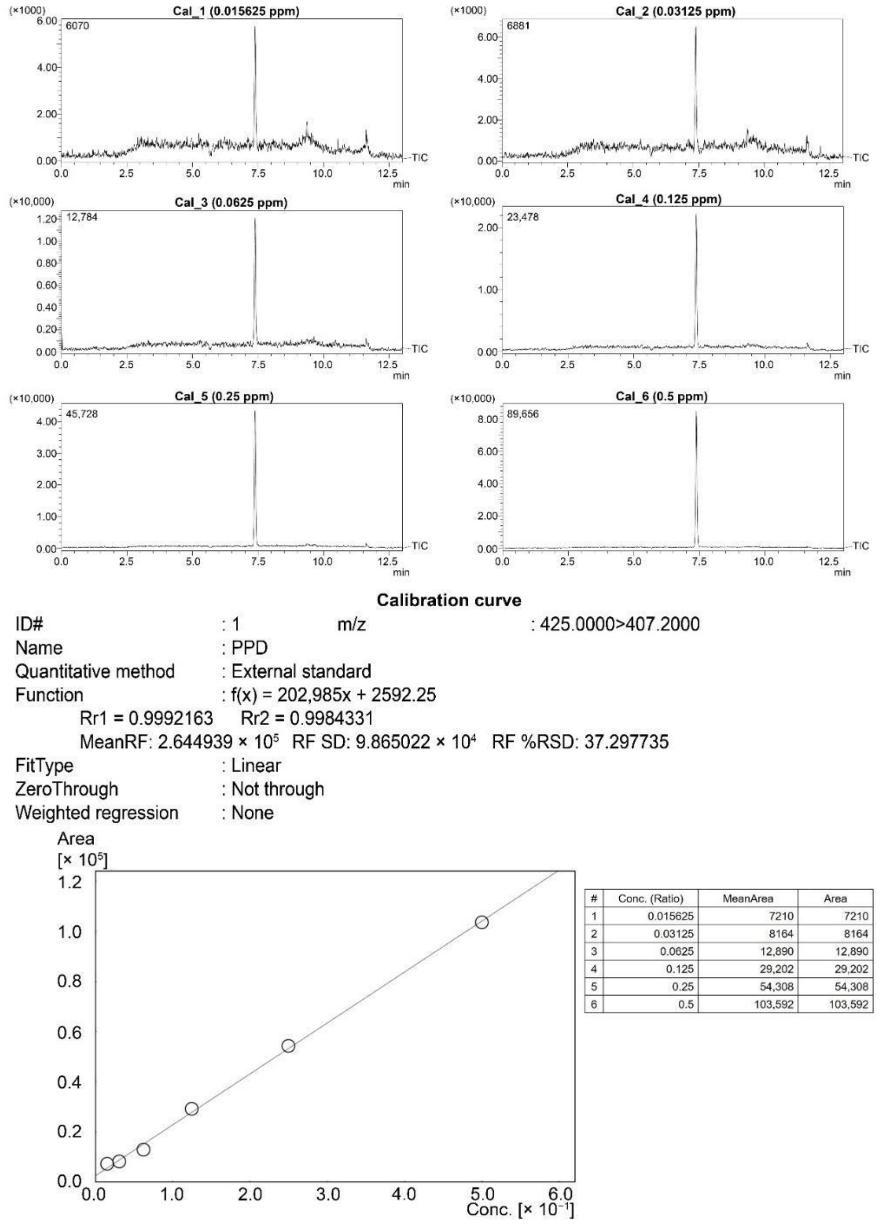

#	Conc. (Ratio)	MeanArea	Area
1	0.015625	7210	7210
2	0.03125	8164	8164
3	0.0625	12,890	12,890
4	0.125	29,202	29,202
5	0.25	54,308	54,308
6	0.5	103,592	103,592

Figure S2. The calibration curve of PPD over the concentration range of 0.0156–0.50000 ppm.

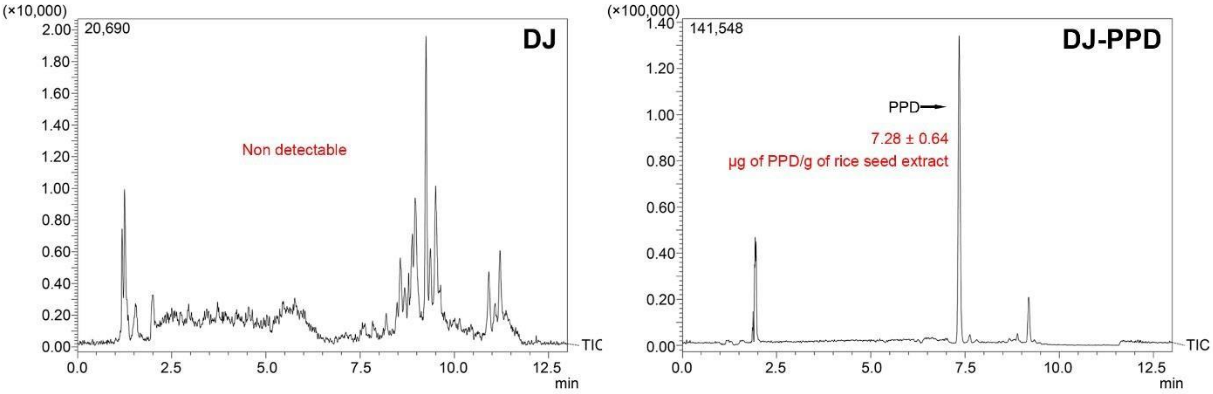

Figure S3. PPD analysis using LC-MS in DJ and DJ-PPD rice seeds. .
